# Longitudinal shifts in oral microbiome composition and metabolic pathways associated with preterm birth

**DOI:** 10.1128/msystems.00184-26

**Published:** 2026-07-10

**Authors:** Mousumi Sarkar, Ankita Maddheshiya, Pragya Tailor, Shankha Nath, Nisha Makkar, GARBH-Ini Study Group, Shinjini Bhatnagar, Sumit Misra, Bapu Koundinya Desiraju, Nitya Wadhwa, Shinjini Bhatnagar, Pallavi Kshetrapal, Souvik Mukherjee

**Affiliations:** 1 Human Microbiome Research Laboratory, Biotechnology Research Innovation Council-National Institute of Biomedical Genomics (BRIC-NIBMG) 214255 , Kalyani, West Bengal, India; 2 Regional Centre for Biotechnology, NCR Biotech Science Cluster 682813 , Faridabad, India; 3 Maternal and Child Health, BRIC-Translational Health Science and Technology Institute, NCR Biotech Science Cluster 682813 , Faridabad, India; 4 Gurgaon Civil Hospital, Gurgaon, Haryana, India; Génomique Métabolique, Genoscope, Institut François Jacob, CEA, CNRS, Université Évry, Université Paris-Saclay, Evry, France

**Keywords:** oral microbiome, term birth, preterm birth, 16S sequencing, shotgun sequencing

## Abstract

Oral dysbiosis in pregnant women with oral diseases has been associated with adverse pregnancy outcomes. However, the inter-individual variability in oral microbiome composition of pregnant women without any oral disease, and its role in preterm birth, has not been studied yet. Here, we have collected saliva from 20 term birth (TB) and 20 preterm birth (PTB) delivering women without any self-reported oral disease at three trimesters (*n* = 120). Microbial DNA was subjected to 16S rRNA gene sequencing for taxonomic classification, and microbial pathways were investigated by PICRUSt2. In a subset of samples, shotgun metagenomic sequencing was done to identify microbial species, their gene families, and their pathways. TB and PTB women were distributed into three distinct oral community types (OCTs). *Haemophilus parainfluenzae* and *Rothia mucilaginosa* were associated with TB and PTB, respectively. The chorismate biosynthesis pathway, essential for folic acid biosynthesis, was significantly enriched in TB, whereas the enterobactin biosynthesis pathway that produces iron chelators (siderophores) was significantly enriched in PTB. The heterolactic fermentation pathway that reduces oral pH was enriched in PTB. Our data suggest that oral microbiome changes might have an impact on birth outcomes in women even without any history of self-reported oral disease during the pregnancy period.

**IMPORTANCE:**

The importance of this study lies in demonstrating that compositional and functional shifts in the oral microbiome are associated with pregnancy outcomes. Using a longitudinal design across three trimesters in an Indian cohort, we show that pregnant women segregate into distinct oral community types with consistent associations to term birth (TB) and preterm birth (PTB). Importantly, the TB-associated microbiome was enriched in taxa and pathways linked to vitamin and amino acid biosynthesis, including chorismate and threonine metabolism, which are critical for fetal growth. In contrast, PTB was associated with pathways related to iron scavenging and acidification of the oral environment, suggesting a metabolically stressed and dysbiotic state. These findings highlight the oral microbiome as a previously underappreciated, modifiable factor in pregnancy outcomes and underscore its potential relevance for early risk stratification and preventive strategies against PTB.

## INTRODUCTION

The oral cavity is home to a conglomerate of more than 500 bacterial species along with fungi, viruses, archaea, and protozoa, and it exhibits high microbial diversity next to the gut (1, 2). It harbors both commensal and pathogenic bacteria that influence oral immunity (2). Different parts of the oral cavity represent a natural habitat for different bacterial species of streptococci, lactobacilli, staphylococci, and corynebacteria (3). It is a unique body site that interacts directly with the external environment, thus acting as a potential entry site for any air or food-borne pathogens. Thus, maintenance of personal oral hygiene is crucial.

A dysbiotic oral microbiome is the probable cause of common oral diseases like dental caries, gingivitis, and periodontitis (1, 4). The altered oral microbiome is also found to be associated with oral cancer (5).

The importance of oral health in pregnant women has recently gained attention in public health research (5). A healthy bacterial community or microbiome is considered a key factor that can balance the oral homeostasis through host-microbiome interactions (2). Hormonal alterations and reduced immunity during pregnancy, aggravated by poor oral hygiene, have been shown to increase the risk of pregnant women developing oral diseases (6). There is a correlation between oral microbiome impairment and adverse pregnancy outcomes such as preterm birth (PTB) (7, 8). Preterm is a condition where the baby is born before 37 weeks of pregnancy. Among them, those that are born due to spontaneous preterm labor without any medical indication are termed spontaneous preterm birth. In India, 27 million babies are born each year, of which 3.5 million babies are born prematurely (9). It has been shown that women suffering from periodontitis in the antenatal period showed a higher risk of developing preeclampsia (8), which is one of the risk factors for PTB. The dysbiotic oral microbiome was strongly associated with periodontal disease (10). However, there is a lack of evidence on the role of the oral microbiome on adverse pregnancy outcomes in pregnant women without any oral disease manifestation. Oral microflora can cause disease in a synergistic manner; thus, looking at a specific taxon may not provide the exact representation of the cooperative phenomena of synergistic interspecific interactions. Rather, the entire microbiome community structure needs to be investigated.

In this study, we hypothesize that pregnant women without any self-reported oral disease conditions show considerable inter-individual variability in the composition and diversity of the oral microbiome that is eventually associated with preterm birth. Such studies are crucial to our understanding of the baseline oral microbiome during pregnancy as well as the role of dysbiosis on birth outcomes in pregnant women with no oral disease manifestation.

This study also aims to characterize the gene families and pathways of the oral microbiome, providing functional insights into its potential role in predisposing women to preterm delivery.

## RESULTS

### Demographic and clinical characteristics of study participants

**Fig 1:**
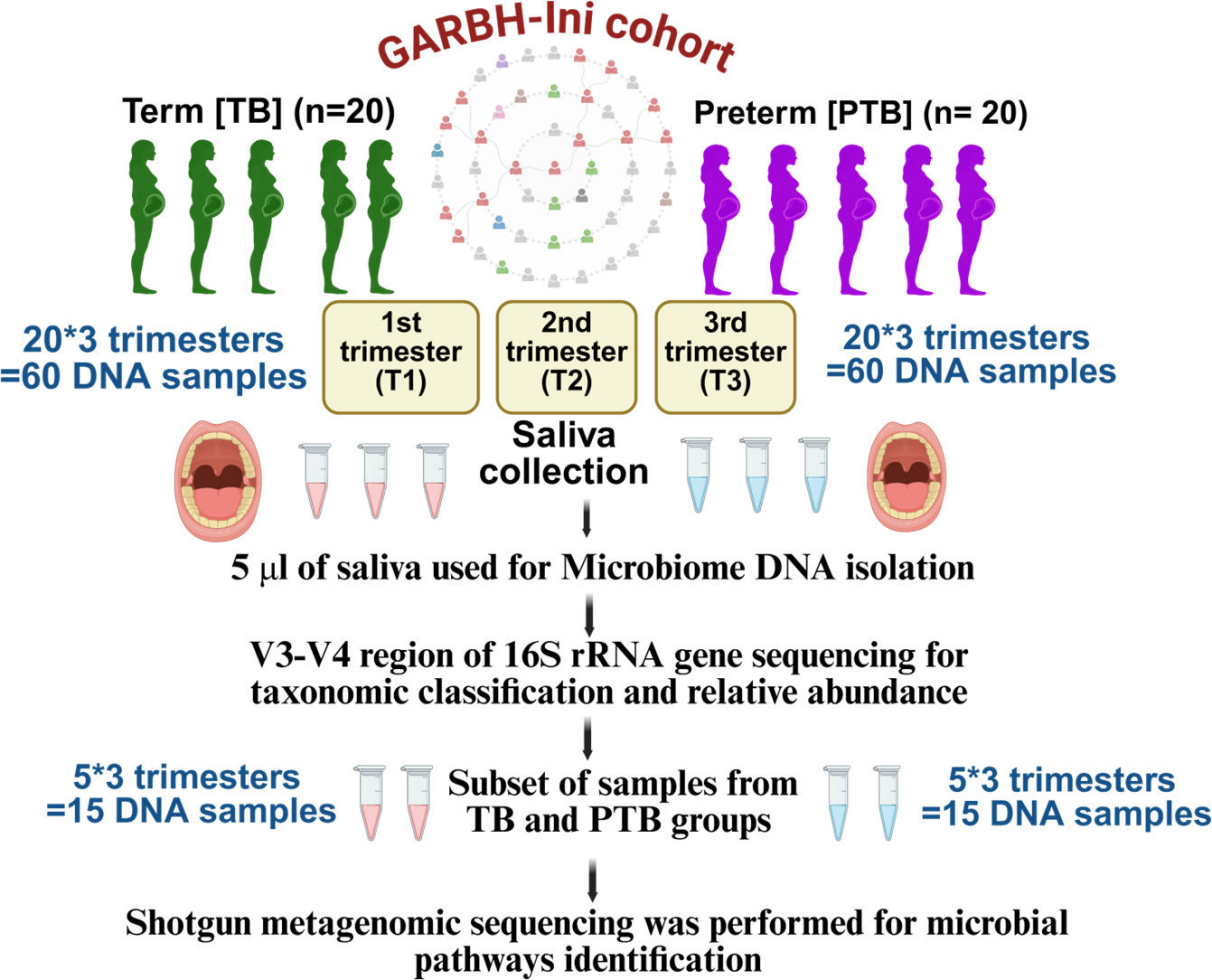
Schematic representation of the oral microbiome study design embedded in the GARBH-Ini pregnancy cohort. A total of 20 term and 20 preterm delivering women were included in this study.

The participants for this study were selected from the GARBH-Ini cohort (Fig. 1), as described in Materials and Methods. The clinical and sociodemographic variables of the study participants are presented in Table 1. Among the study participants, the literacy rate was 94% for TB and 73% for PTB groups. The housing conditions and source of drinking water were similar between the two groups. Average periods of gestation (POG) at delivery in TB and PTB groups were 38.92 (38.04–40.00) weeks and 35.71 (33.64–36.29) weeks, respectively. Median maternal age at enrollment was 21 years in TB and 22 years in PTB participants. Clinical characteristics like body mass index (BMI) for term and preterm groups was 19.76 (18.4–22.31) and 21.85 (18.99–23.69), respectively. Approximately 80% of the women (*n* = 16) in the term and 65% (*n* = 13) in the preterm groups had vaginal deliveries. Neonatal characteristics show that the median weight for term-born and preterm-born infants was 26,00 g (2,500–3,073 g) and 2,196 g (1,900–2,608 g), respectively. Feto-placental weight in the TB and PTB groups was 5.62 g (5.06–6.35 g) and 5.42 g (4.09–6.58 g), respectively. Nearly 50% of the infants were male and the remaining 50% were female in the TB group, whereas in the PTB group, 43% of the infants were male and 56% were female.

**TABLE 1:** Sociodemographic and clinical characteristics of study participants^*b*^

Variable	Term, *n* = 20	Preterm, *n* = 20
Sociodemographic variables		
Education, *n*, %		
Illiterate-primary school	3/20, 15%	6/20, 30%
Middle-Hr. sec school	13/20, 65%	12/20, 60%
Graduate and above	4/20, 20%	2/20, 10%
Occupation status of mother, *n* (%)		
Unskilled worker	1/20 (5%)	0/20 (0%)
Unemployed	19/20 (95%)	20/20 (100%)
Family income (Rs)	12,000 (9,375–15,750)	15,000 (10,000–28,000)
Type of house, *n* (%)		
Kuccha	1/20 (5%)	1/20 (5%)
Pucca	17/20 (85%)	19/20 (95%)
Semi pucca	2/20 (10%)	0/20 (0%)
Source of drinking water^*a*^		
Safe water	20/20 (100%)	19/20 (95%)
Unsafe water	0/20 (0%)	1/20 (5%)
Toilet facility		
Flush or pour flush toilet, *n* (%)	19/20 (95%)	20/20 (100%)
Bucket latrine, *n* (%)	1/20 (5%)	0/20 (0%)
Maternal characteristics		
Age (yrs)	21 (20–23)	22 (21–24)
Gestational age at delivery (wks)	38.86 (38.04–40.00)	35.71 (33.64–36.29)
BMI (kg/m^2^)	19.76 (18.29–22.31)	21.85 (18.99–23.69)
Smoking status		
Yes, *n* (%)	0/20 (0%)	0/20 (0%)
No, *n* (%)	20/20 (100%)	20/20 (100%)
Alcohol consumption status, *n* (%)		
Yes	0/20 (0%)	0/20 (0%)
No	20/20 (100%)	20/20 (100%)
Tobacco chewing status, *n* (%)		
Yes	0/20 (0%)	0/20 (0%)
No	20/20 (100%)	20/20 (100%)
Mode of delivery, *n* (%)		
Cesarian	4/20 (20%)	7/20 (35%)
Vaginal	16/20 (80%)	13/20 (65%)
Parity, *n* (%)		
0	14/20 (70%)	6/20 (30%)
1	6/20 (30%)	14/20 (70%)
Neonatal characteristics		
Infant wt^*c*^ (*P-*value 0.01) (g)	2,600 (2,500–3,073), *n* = 19/20	2,196 (1,900–2,608), *n* = 18/20
Placental wt (g)	520.8 (391.5–565.9), *n* = 16/20	393.7 (319.2–538.9), *n* = 12/20
Feto-placental wt	5.62 (5.06–6.35), *n* = 15/20	5.42 (4.09–6.58), *n* = 12/20
Gender		
Male, *n* (%)	9/18, 50%	7/16, 43.75%
Female, *n* (%)	9/18, 50%	9/16, 56.25%

^*a*^ Safe water includes bottled water or piped water into the residence. Unsafe water includes sources from open wells.

^*b*^ Data expressed as median (IQR) or number (percentage), Counts as (*n*, %) for continuous and categorical outcome variables, respectively.

^*c*^ *P-*value < 0.05. Statistical analysis by unpaired *t*-test using GraphPad Ver 10.

### Oral microbial diversity during pregnancy

**Fig 2:**
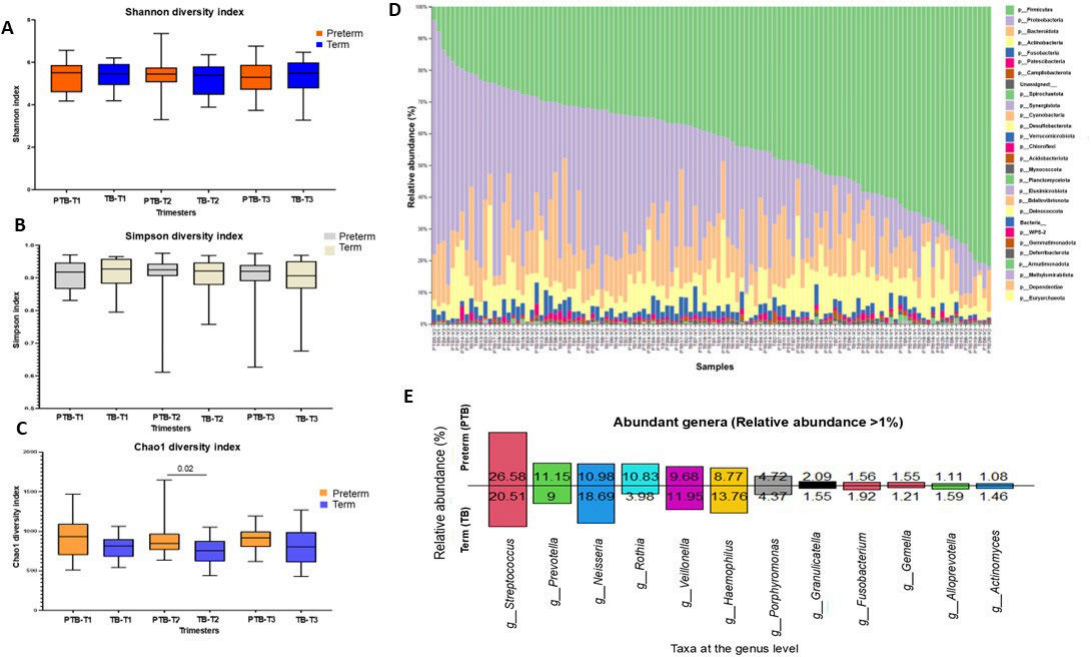
(**A**) Shannon diversity index, (**B**) Simpson diversity index, and (**C**) Chao1 diversity index in the TB and PTB groups. (**D**) Bar plot showing the relative abundance of the phyla present in the oral environment. *X*-axis denotes the individual samples. *Y*-axis denotes the relative abundance (%) of the phylum. Colors denote different phyla. (**E**) The dual bar plot showing the core genera and their relative abundance in TB and PTB groups. The *Y*-axis denotes the relative abundance (%) of the core genus; different colors denote different genera that are mentioned in the *X*-axis, and the height of the bar represents the relative abundance of the specific taxa mentioned in the *X*-axis.

Among the alpha diversity measures, except Chao1, the other two indices, that is, Shannon and Simpson, were found to be similar across both groups (Fig. 2A through C). The Chao1 diversity measure was comparatively lower in TB compared to PTB in all trimesters, with significant differences observed at the second trimester (PTB: 913.69 ± 245.69 at T2 and TB: 739.41 ± 165.16 at T2, *P*-value = 0.02) (Fig. 2C). However, within-group comparisons in the TB and PTB groups longitudinally did not show any significant differences in alpha diversity indices across trimesters (Fig. 2A through C).

To study the inter-individual diversity in the TB and PTB groups, Jaccard (PERMANOVA: *P*-value = 0.43) and Bray-Curtis (PERMANOVA: *P*-value = 0.35) indices (the beta diversity indices) were estimated. However, no significant differences were observed in both diversity indices across the PTB and TB groups (Fig. S1).

### Core microbiome from saliva samples of pregnant women

A total of 26 phyla and 803 genera were obtained from 52,487 amplicon sequence variant (ASV) sequences. Only those taxa were selected as core that showed ≥0.5% relative abundance and were present in at least 50% of individuals in any of the groups at any trimester. Among the 26 phyla, 7 were selected as core, which together constituted ~99% of the total microbial abundance. At the phylum level, Firmicutes (TB_T1: 42.86 ± 13.78; PTB_T1: 43.71 ± 13.97) was the most abundant, followed by Proteobacteria (TB_T1: 31.55 ± 16.15; PTB_T1: 26.67 ± 14.30) (Fig. 2D).

At the genus level, 19 genera were selected as core, comprising ~94% of the total abundance. The results showed that the genus *Streptococcus* was the most dominant taxon in both TB and PTB groups in all trimesters. Other predominant genera were *Neisseria, Rothia, Prevotella, Haemophilus, Veillonella, Porphyromonas, Fusobacterium*, *Gemella,* and *Alloprevotella* (Fig. 2E and Table S1).

In this study, 32 species were identified as core species, constituting ~91% of the total microbiome abundance. Among them, 10 species belonged to the *Streptococcus* genus, with *Streptococcus symci* being the most abundant species. Other abundant species were *Neisseria perflava, Haemophilus parainfluenzae, Rothia mucilaginosa, Prevotella melaninogenica,* and *Neisseria subflava* (Table S2).

### Dynamic nature of oral microbiome-oral community type (OCT) at the genus level

**Fig 3:**
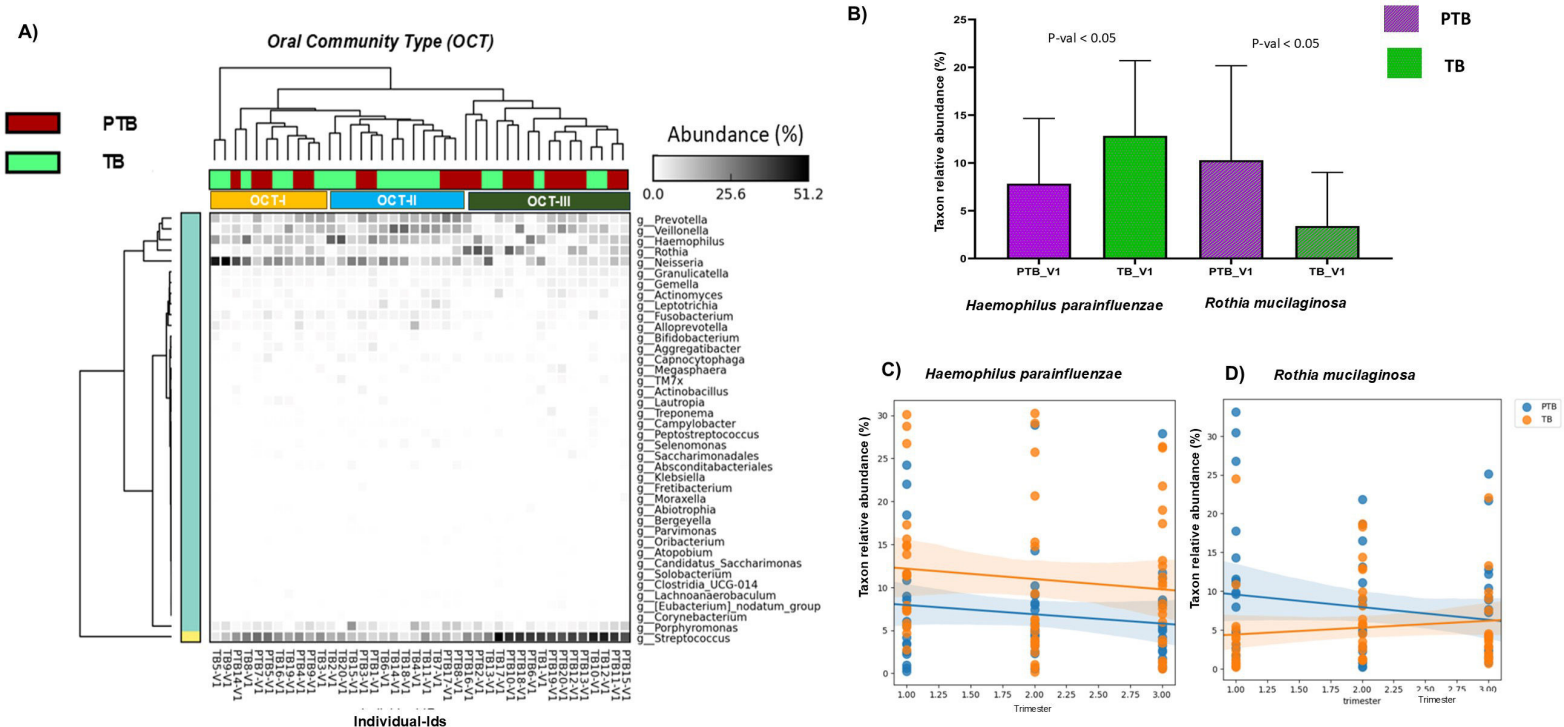
(**A**) The dendrogram on hierarchical clustering representing the OCT based on oral microbial taxa composition. *Y*-axis denotes the core species; *X*-axis denotes the individual samples. The three different OCTs are denoted by three different colors: OCT-I by yellow, OCT-II by blue, and OCT-III by green. The color bar at the upper right corner represents the relative abundance from the lowest to the highest value. (**B**) The bar plot shows the differences in relative abundance of *Haemophilus parainfluenzae* and *Rothia mucilaginosa* in term and preterm samples at T1. *Y*-axis represents the relative abundance (%); *X*-axis represents the bacterial species in TB and PTB groups in the first trimester. (**C**) Results of Linear Mixed Effect (LME) analysis showing significant longitudinal variations in relative abundances of microbial taxa across trimesters within TB and PTB groups. *Y*-axis denotes the relative abundance (%) of the species; *X*-axis denotes the different trimesters. PTB samples are represented by blue color; TB samples are represented by orange color. Each dot in the figure represents the individual level relative abundance of the mentioned species.

Different body parts have unique signature microbiota, such as *Bifidobacterium* in the gut and *Lactobacillus* in the vagina, but the signature taxon of the oral cavity is not well understood (8), being mostly populated by diverse bacterial communities that inhabit the tongue, buccal mucosa, supragingival and subgingival surfaces of the teeth, and soft and hard palates. In our study, using hierarchical clustering on microbiome data, three distinct groups were observed, dominated by different oral taxa, and are classified as OCT. The OCT-I was dominated by the genus *Neisseria*, OCT-II was dominated by mixed taxa (*Neisseria*, *Prevotella*, *Haemophilus,* and *Veillonella*), and OCT-III was dominated by the genus *Streptococcus* (Fig. 3A). This shows that the oral microbiome is very dynamic in nature, and the establishment of a signature taxon is very challenging. Almost 32% (*n* = 13) of women were in OCT-I, 25% (*n* = 10) were in OCT-II, and 43% (*n* = 17) were in OCT-III. This suggests that the *Streptococcus* OCT III is the most commonly observed OCT among PTB (50%; 10/20) as well as TB (35%; 7/20) women (Table 2).

**TABLE 2:** OCT distribution in TB and PTB groups

OCT type	OCT-dominated taxa	Total no. of women (*n* = 40)	No. of TB (*n* = 20)	TB %	No. of PTB (*n* = 20)	PTB %	*P*-value
OCT-I	*Neisseria*	13 (32%)	8	40%	5	25%	0.034
OCT-II	Mixed	10 (25%)	5	25%	5	25%	1
OCT-III	*Streptococcus*	17 (43%)	7	35%	10	50%	0.045

### Differential oral microbiome profiles in term and preterm delivering mothers

Alteration of oral microbiome was observed between TB and PTB in different trimesters of pregnancy. *H. parainfluenzae* was found to be higher in every trimester in the TB group compared to the PTB group, with significantly higher abundance at T1 (TB_T1: 12.83 ± 7.86, PTB_T1: 7.18 ± 6.82; *P*-value: 0.02). *Rothia mucilaginosa* was found to be higher in PTB at first and third trimesters compared to TB, with significantly higher abundance at T1 (TB_T1: 3.40 ± 5.61, PTB_T1: 10.29 ± 9.88; *P*-value: 0.002) (Fig. 3B).

The most abundant species in our study, *Streptococcus symci,* was found to be similar across the TB and PTB groups, but interestingly, the other species of *Streptococcus* showed a comparatively higher abundance in TB compared to the PTB group with significant dysbiosis observed at the second trimester. At T2, *Streptococcus salivarius* (TB_T2: 1.99 ± 2.2, PTB_T2: 0.56 ± 0.53; *P*-value: 0.02), *Streptococcus peroris* (TB_T2: 0.72 ± 0.83, PTB_T2: 0.20 ± 0.24; *P*-value: 0.03), and *Streptococcus ilei* (TB_T2: 2.0 ± 3.68, PTB_T2: 0.88 ± 1.42; *P*-value: 0.02) were found to be significantly higher in the TB group. In T3, oral taxa like *Haemophilus sputorum* (TB: 0.51 ± 0.63, PTB: 0.20 ± 0.32; *P*-value: 0.03) and *Aggregatibacter aphrophilus* (TB: 0.54 ± 0.62, PTB: 0.16 ± 0.19; *P*-value: 0.0004) were found to be significantly higher in TB. It was observed that few oral taxa were associated with the BMI of the pregnant women both in the TB and PTB groups (Supplemental material and Fig. S2)

### Longitudinal shift of the oral microbiome

LME-based analysis was performed as mentioned in Materials and Methods, using q2-longitudinal (11). The effects of both birth types (PTB/TB) and gestational time (trimesters) were significant for *H. parainfluenzae*, which showed higher relative abundance in the TB from the first to the third trimester of the gestational period compared to PTB. The mean relative abundance of *Rothia mucilaginosa* was found to be higher longitudinally in PTB compared to TB (Fig. 3C). These two bacteria were found to be key contributors, achieving a prediction accuracy of approximately 75% for distinguishing between TB and PTB groups (Supplemental material and Fig. S3).

### Discriminating microbial pathways of oral microbiome between term and preterm delivering women

**Fig 4:**
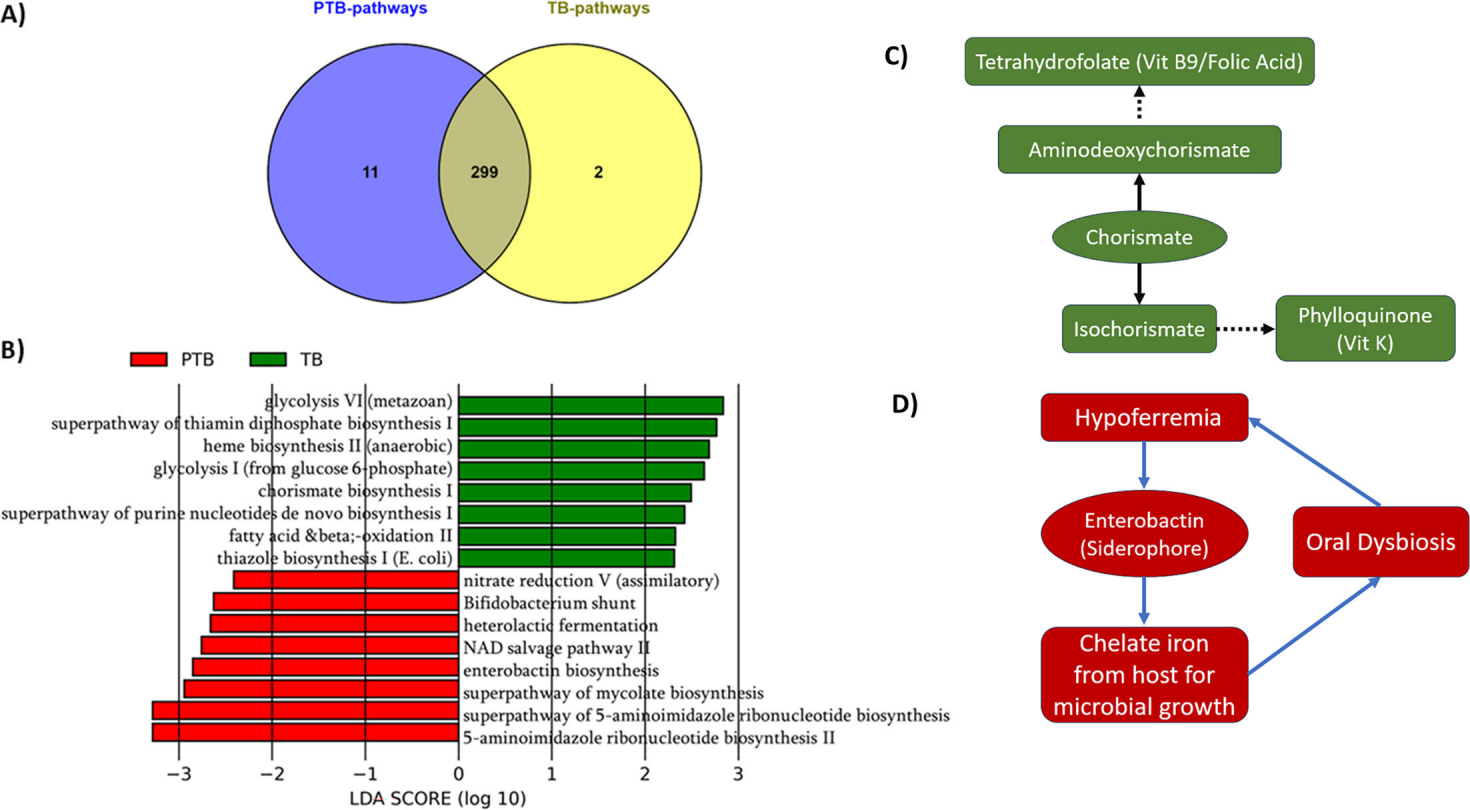
Common and significantly altered pathways in the TB and PTB groups in different trimester time points. (**A**) Venn diagram showing the total number of pathways present in TB and PTB samples. (**B**) The LDA plot showing the differentially enriched pathways between TB and PTB groups at the first trimester (T1). (**C**) Schematic representation of the involvement of Chorismate in the biosynthesis of Vit B9 and K. (**D**) Schematic representation of the involvement of enterobactin in oral dysbiosis.

Shotgun metagenomic sequencing identified 312 pathways at the community level. Out of the total pathways obtained from 16S data by PICRUST2 analysis, ~205 overlapped with those identified from shotgun metagenomic sequencing data. We have included these common pathways for further statistical analysis. The chorismate biosynthesis-I (precursor of folate) and fatty acid & beta oxidation-II pathways were found to be significantly higher in the TB group, whereas the enterobactin biosynthesis and heterolactic fermentation pathways were significantly higher in the PTB group from shotgun data at T1 (Fig. 4). This was also similar to the results obtained by Picrust2 analysis of 16S data where the enterobactin biosynthesis and D-galactarate degradation pathways in the PTB group and the biotin biosynthesis, fatty acid, and beta-oxidation-I pathways in the TB group were found to be enriched at T1.

Species-level resolution of the pathways from the shotgun data revealed that the *H. parainfluenzae* and *Streptococcus oralis* had higher abundance of the chorismate biosynthesis-I pathway in the TB group at T1. Chorismate is a secondary metabolite of bacteria that participates in folic acid (Vit B9) and Vit K biosynthesis (Fig. 4C).

In the TB group, a higher abundance of *H. parainfluenzae* was maintained throughout the pregnancy. Superpathway of L-threonine biosynthesis of *H. parainfluenzae* was significantly enriched in the TB group at T1 and T3. *S. salivarius,* which was significantly higher in TB at T2, showed higher abundance of biotin biosynthesis, fatty acid, and beta-oxidation-I, as well as folate transformation, aromatic amino acid, and L-valine biosynthesis pathways.

In the PTB group, *Rothia mucilaginosa* was present in higher abundance throughout pregnancy. We observed superpathway of 5-aminoimidazole ribonucleotide biosynthesis pathway of *R. mucilaginosa* to be significantly enriched in the PTB group at T1.

## DISCUSSION

The oral microbiome, a diverse assemblage of bacteria inhabiting the oral cavity, is essential for sustaining both oral and systemic health (3). Oral dysbiosis can result in dental caries, periodontitis, halitosis, systemic diseases including cardiovascular diseases, diabetes, respiratory infections, and adverse pregnancy outcomes (2, 12, 13). Therefore, maintaining the equilibrium of the oral microbiome is essential for managing and preventing various systemic disorders.

The current study is a longitudinal nested case-control study embedded in the GARBH-Ini cohort from India (14), focusing on oral dysbiosis and its role in adverse pregnancy outcomes like PTB. To the best of our knowledge, this is the first study that characterizes the oral microbiome associated with PTB in Indian women. Previous oral PTB studies have largely focused on periodontal disease-enriched or high-risk obstetric settings, where elevated periodontal pathogens such as *P. gingivalis*, *T. forsythia*, *P. intermedia,* and *F. nucleatum* in subgingival plaque or saliva samples were associated with preterm delivery (10, 15). In contrast, our longitudinal Indian cohort was not centered on clinically diagnosed periodontitis but identified broader pregnancy outcome-associated shifts in the oral microbiome and its functional potential, linked to term birth and dysbiotic signatures associated with PTB. Our study highlights that the oral microbiome composition is dominated either by Firmicutes or Proteobacteria at the phylum level. At the genus level, the oral microbiome of pregnant women was seen to be dominated by *Neisseria* (OCT-I); *Neisseria, Prevotella, Veillonella,* and *Haemophilus* (OCT-II), and *Streptococcus* (OCT-III) genera.

In our study, an association was observed between term birth and the presence of *S. salivarius*. Our data showed that *S. salivarius* harbors biotin biosynthesis, folate transformation, aromatic amino acid, and L-valine biosynthesis pathways. A previous study has highlighted that *S. salivarius* exhibited probiotic activity by producing bacteriocin molecules (16). This less acid-tolerant species, *S. salivarius,* produces large amounts of alkaline substance maintaining the acid-base balance of the oral cavity (17).

*Prevotella, Rothia, Gemella, Capnocytophaga,* and *Neisseria* are considered commensals of the oral cavity (18). The current study showed that the abundance of *Prevotella, Rothia, Gemella, Capnocytophaga,* and *Neisseria* differed in term and preterm delivering women. A comparatively higher abundance of *R. mucilaginosa* was always maintained in PTB samples, but in contrast, a higher abundance of *H. parainfluenzae* was associated with TB samples throughout the pregnancy. *R. mucilaginosa* is an opportunistic bacterium in the oral cavity that causes bacteremia in immunocompromised individuals (19, 20). A study on pediatric dental caries showed that *R. mucilaginosa* emerged as one of the key biomarkers for the prediction of early childhood caries onset (21). On the other hand, *H. parainfluenzae* is an oral commensal that was found to be more prevalent in healthy individuals compared to those with periodontitis, with its abundance correlating with a healthier oral status (22, 23).

The enterobactin biosynthesis pathway was found to be enriched in the PTB group. This pathway can produce selective iron chelators (siderophores) in response to iron deficiency for microbial growth (Fig. 4D) (24). Increased siderophores in the placenta have been reported to be related to iron deficiency anemia in PTB and pre-eclampsia (25). The current study showed high abundance of fatty acid and beta-oxidation-II pathway in term samples. Fatty acids are components of cell membranes or dietary lipids (26). Beta-oxidation plays a crucial role in maintaining homeostasis by utilizing the excess lipid or fatty acid (27), and by utilizing it, beneficial oral bacteria can potentially colonize within host tissues by outcompeting the pathogenic bacteria in the oral cavity (28, 29).

Heterolactic fermentation, a metabolic pathway utilized by certain *Lactobacillus* to ferment carbohydrates into lactic acid, acetic acid, and ethanol, was found to be significantly higher in PTB samples at T1 and T3. This can change the oral pH and thus may lead to microbiome dysbiosis (30).

The chorismate biosynthesis pathway was found to be significantly higher in term delivering mothers in T1. Chorismate is an essential metabolite for the synthesis of folic acid and vitamins E and K (Fig. 4C). It is found to be harbored by *H. parainfluenzae* and *S. oralis*, both TB-associated taxa. Folic acid, vit K, and vit E are essential for the growth and development of the fetus, and its deficiency is associated with gestational anemia in pregnant women (31).

Superpathway of L-threonine biosynthesis of *H. parainfluenzae* was significantly predominant in the TB group at T1 and T3. The study showed that at late stages of pregnancy, the requirement of threonine increased by 55% compared to the early stages (32). We also observed that the superpathway of 5-aminoimidazole ribonucleotide biosynthesis of *R. mucilaginosa* was significantly enriched in the PTB group at T1. This pathway is responsible for the biosynthesis of purine nucleotides in bacteria that helps in DNA, RNA, and protein synthesis (33), which might influence the growth of pathogenic bacteria.

In this study, the significantly different microbial taxa were identified in both PTB and TB. Novel biological insights have been obtained on the functional potential of the oral microbial taxa that may support pregnancy maintenance and facilitate the overall development of the fetus in term delivering mothers. The current study provides valuable insight into the role of the oral microbiome in pregnancy outcomes. This study emphasizes the need for a healthy oral environment during pregnancy and can be considered a public health strategy addressing PTB in the future.

Our study is performed with a modest number of pregnant women delivering term and preterm babies. The random forest prediction is also performed on these 40 samples. Hence, the result of this study provides first-hand information about the importance of the oral microbiome in birth outcomes, which needs to be validated in larger cohorts for global generalizability. The oral-health assessment was performed by questionnaire method without oral clinical examination, which is a major limitation of this study. Diet practice has a considerable impact on oral microbiome composition; however, in the current study, due to the unavailability of information on food habits, we could not correlate it with the microbial data. This study provides novel insights into the association between oral microbiome composition and pregnancy outcomes in Indian women through a longitudinal nested case-control study. Our findings highlight that the oral microbiome undergoes significant changes during pregnancy, with distinct microbial and functional signatures observed between term birth and preterm birth delivering women. Our data suggest that the oral microbiome might have an impact on birth outcomes.

## MATERIALS AND METHODS

### Study design and study population

The current study was designed as a nested case-control study within the ongoing GARBH-Ini cohort. GARBH-Ini (the Interdisciplinary Group for Advanced Research on Birth Outcomes-DBT India Initiative) is an ongoing prospective observational hospital-based pregnancy cohort at Gurugram, Haryana, India. The study was initiated with the hypothesis that time-series data collected on multidimensional characteristics—including clinical, imaging, environmental, genomic, epigenomic, metagenomic, proteomic, and metabolomic data during pregnancy—will identify individuals at a high risk of delivering preterm (<37 completed weeks of gestation).

Our study was approved by the ethics committees of the Gurugram Civil Hospital, Gurugram; BRIC-Translational Health Science and Technology Institute, Faridabad; and BRIC-National Institute of Biomedical Genomics, Kalyani; and the study adhered to the tenets of the Declaration of Helsinki as part of the overall GARBH-INi cohort objectives. Written informed consent was obtained from the participants. Details of enrollment and biospecimen collection were as described in a previous study by Bhatnagar et al. (14). The pregnant women with no medical complications during pregnancy who delivered live singleton newborns spontaneously and had no congenital abnormalities were included in this study. Women with any history of antibiotic usage within 7 days prior to sampling were excluded. They were included in the study following a self-assessment of their oral health, which screened for conditions like dental caries and periodontitis through a questionnaire method, without any clinical investigation. The criteria guiding this participant-reported screening were based on the methodology described by Myers-Wright et al. (34). Cases were women who had spontaneous preterm delivery, and controls were women who delivered at term, >37 weeks of gestation. A total of 40 women were selected, of whom 20 delivered at term and 20 delivered at preterm. Saliva samples collected at first trimester (<14 weeks), second trimester (18–20 weeks), and third trimester (26–28 weeks) from the enrolled participants, respectively, were processed for DNA isolation and metagenomic sequencing study (Fig. 1).

### DNA isolation and next-generation sequencing

DNA isolation was performed using QiAmp BiOstic Bacteremia DNA Kit (35). Microbiome sequencing of the V3-V4 region of the 16S rRNA gene was carried out for all the 20 TB and 20 PTB samples for all trimesters (*n* = 120), and those samples with optimum DNA concentration ≥0.2 ng/µL were carried forward for shotgun metagenomic sequencing using the NovaSeq6000 platform following 2 × 250 bp paired-end chemistry.

### 16S data analysis

Raw sequence reads were quality-filtered and classified into ASVs using QIIME2 (36), and representative sequences were aligned with SILVA-v.138 (37) reference for taxonomic classification based on VSEARCH consensus taxonomy classifier (38, 39). For species identification, the representative sequences were aligned against the NCBI 16S rRNA microbial database by BLAST. The species were assigned only when the query fulfilled the BLAST identity ≥99% (35, 38). ASV feature table was further used for the estimation of alpha and beta diversity indices. For the functional prediction of 16S data (*n* = 120), Picrust2 was used (40, 41). For Picrust2 analysis, ASV and Biome files were utilized as input. Multiple assignments of exact sequence variants were carried out with the help of HMMER (http://www.hmmer.org/), which was then followed by evolutionary placement-ng (EPA-ng) and Genesis Applications for Phylogenetic Placement Analysis (gappa) omics. Pathway prediction was done using the pathway_pipeline.py script, which assigns EC values to MetaCyc reactions and KO abundances in KEGG pathways. The predicted pathways were also validated by shotgun metagenomic sequencing in a subset of samples. A total of 30 microbiome DNA (*n* = 30) from 15 term (5 term women × 3 trimesters) and 15 preterm (5 preterm women × 3 trimesters) women with optimum DNA concentration (≥0.2 ng/µL) were subjected to shotgun metagenomic sequencing. The subset of term and preterm samples were matched based on the same age group and the same month of delivery.

### Shotgun metagenomic data analysis

Raw reads were mapped to the human reference genome (hg19) using Bowtie2 (42) to identify and remove human reads. Read pairs <36 bases, quality value (QV) <25, were discarded using Trimmomatic tool (43). Quality-filtered reads were mapped by MetaphlAn2 to a database of clade-specific marker genes for taxonomic profiling (44). For functional annotation at community total and species-resolved gene families, reads were mapped to the ChocoPhlan pangenome database by HUMAnN2 (45, 46). Unmapped reads were further mapped to the UniRef-based protein sequence database (UniRef-90) (47) for translational search. HUMAnN2 was used to reconstruct pathways and estimate community total and species-resolved microbial pathways based on the MetaCyc database (48). The “unintegrated/unmapped/unknown/ungrouped” pathways were removed using the “humann2 renorm table” script before downstream analysis. Only those microbial pathways were described in the result section that were either significantly discriminant between term and preterm or uniquely present in one group.

### Statistical analyses

Sociodemographic parameters were compared between term and preterm delivering mothers using χ^2^ test for categorical variables and Wilcoxon rank-sum test for continuous variables. Differences in diversity indices and oral microbiome composition between term and preterm groups were estimated by Wilcoxon rank-sum test (two-tailed) using the R command (wilcox.test, paired = FALSE) at each trimester separately. Analysis of variance (ANOVA) test was performed for comparing the three time points (T1, T2, and T3) within each group. Correlations between pairs of predominant oral taxa at the species level were evaluated using non-parametric Spearman’s rank correlation test in R (cor.test, method = spearman). LME analysis was performed to understand the longitudinal shift of the oral microbiome where the birth type (term or preterm) and gestational time (first, second, or third trimesters) were used as fixed effects and maternal age as a random effect (11). Discriminating pathways of term and preterm groups were identified using linear discriminant analysis (LDA) effect size (LEfSe) (49). Random forest analysis was performed using species abundance data to predict preterm and term outcomes of pregnancy. Random Forest analysis was performed with the help of the R packages “caret” (https://topepo.github.io/caret/) (50) and “randomForest,” where 80% of the data were used as training and the rest 20% of the data as test sets (see Supplemental material for codes). The STORMS checklist can be accessed through this link: https://shorturl.at/ZBYHF.

## Data Availability

The sequencing data generated in this study have been deposited in the Indian Biological Data Center, Indian Nucleotide Data Archive, under accession number INRP000685 and are publicly available at https://ibdc.dbt.gov.in/inda/home.

## SUPPLEMENTAL MATERIAL

Fig. S1 msystems.00184-26-s0001.tif PCoA plots.

Fig. S2 msystems.00184-26-s0002.tif Significant correlation plots between core microbial species and BMI.

Fig. S3 msystems.00184-26-s0003.tif Results of the random forest analysis.

Supplemental Material msystems.00184-26-s0004.docx Supplemental methods, tables, and figure legends.

ASM does not own the copyrights to Supplemental Material that may be linked to, or accessed through, an article. The authors have granted ASM a non-exclusive, world-wide license to publish the Supplemental Material files. Please contact the corresponding author directly for reuse.
