# Supplementary material for: Longitudinal shifts in oral microbiome composition and metabolic pathways associated with preterm birth
Source: mSystems. 2026 Jul 10;11(8):e00184-26. doi: 10.1128/msystems.00184-26 (PMC13483446; doi:10.1128/msystems.00184-26)
Supplement: Supplementary file 1 [file msystems.00184-26-s0004.docx]

**Supplementary appendix I:**

**BMI is correlated with the oral microbiome abundance during pregnancy:**

Previous studies have shown that, certain oral bacteria are associated with the weight gain in humans, supporting speculations that oral microbial flora may harbour such pathways leading to obesity. We analysed the correlation between BMI of pregnant women and the oral microbiome abundance in TB and PTB separately. Interestingly, we could identify few microbial species that were significantly correlated with BMI in our study population, but the correlation pattern differed between TB and PTB groups. In PTB, significant negative correlation was observed between BMI and oral bacteria *Porphyromonas endodontalis* (ρ-value: -0.44, p-value: 0.04), whereas a significant positive correlation was observed for *Haemophilus sputorum* (ρ-value: 0.50, p-value 0.02). In case of TB group, we have observed a significantly positive correlation between BMI and oral taxa like *Aggregatibacter aphrophilus* (ρ-value: 0.45, p-value: 0.04), *Haemophilus sputorum* (ρ-value: 0.47, p-value: 0.03).

**Supplementary appendix II:**

**Prediction of term and preterm birth at the initial period of pregnancy:**

To address the challenge of high dimensionality in a small sample size, random forest (RF) analysis was conducted using the relative abundances of microbial species that were significantly different between groups. The primary aim of the RF analysis was to evaluate the discriminatory power of *Haemophilus* *parainfluenzae* and *Rothia* *mucilaginosa*, which were identified as being associated with TB and PTB outcomes at the first trimester timepoint and consistently higher in all the trimesters in the respective groups. The importance plot from the RF classifier demonstrated that *Haemophilus parainfluenzae* (associated with TB) and *Rothia mucilaginosa* (associated with PTB) were key contributors, achieving a prediction accuracy of approximately 75% for distinguishing between TB and PTB groups. These findings underscore the potential role of these two oral taxa, particularly during the first trimester, in distinguishing TB and PTB groups during the early stages of pregnancy.

**Supplementary information**

**Supplementary Table S1:**

**The table shows the relative abundance (%) of the core genera in term (TB) and preterm (PTB) groups at each of the trimesters.**

| **Genus** | **Average (%) and Standard deviation (±)** | | | | | | | | | | | |
| --- | --- | --- | --- | --- | --- | --- | --- | --- | --- | --- | --- | --- |
|  | **Average-PTB-T1**  **(%)** | **(±) SD** | **Average-TB-T1 (%)** | **(±) SD** | **Average-PTB-T2 (%)** | **(±) SD** | **Average-TB-T2 (%)** | **(±) SD** | **Average-PTB-T3**  **(%)** | **(±) SD** | **Average-TB-T3 (%)** | **(+) SD** |
| *g__Veillonella* | 9.68 | 6.28 | 11.95 | 9.03 | 11.24 | 15.08 | 8.32 | 7.61 | 6.42 | 7.43 | 8.18 | 7.78 |
| *g__Haemophilus* | 8.77 | 6.95 | 13.76 | 8.34 | 8.13 | 6.27 | 10.86 | 10.81 | 6.48 | 6.59 | 11.95 | 9.17 |
| *g__Prevotella* | 11.15 | 6.68 | 9.00 | 5.38 | 6.76 | 6.06 | 7.46 | 5.42 | 6.15 | 6.32 | 6.86 | 6.10 |
| *g__Actinomyces* | 1.08 | 1.18 | 1.46 | 1.50 | 1.15 | 0.86 | 1.62 | 1.34 | 1.46 | 1.98 | 1.01 | 1.08 |
| *g__Neisseria* | 10.98 | 8.85 | 18.69 | 13.07 | 22.99 | 16.96 | 16.63 | 12.81 | 16.63 | 12.46 | 17.89 | 15.94 |
| *g__Rothia* | 10.83 | 10.14 | 3.98 | 5.90 | 7.22 | 6.62 | 7.73 | 5.74 | 7.50 | 7.03 | 5.68 | 5.24 |
| *g__Corynebacterium* | 0.17 | 0.12 | 0.32 | 0.20 | 0.56 | 0.50 | 0.19 | 0.14 | 0.27 | 0.22 | 0.22 | 0.15 |
| *g__Campylobacter* | 0.25 | 0.19 | 0.41 | 0.26 | 0.43 | 0.29 | 0.51 | 0.53 | 0.37 | 0.33 | 0.71 | 0.79 |
| *g__Streptococcus* | 26.58 | 11.29 | 20.51 | 13.49 | 21.43 | 16.73 | 31.89 | 18.73 | 35.91 | 20.17 | 32.02 | 17.21 |
| *g__Alloprevotella* | 1.11 | 1.20 | 1.59 | 2.85 | 0.93 | 0.88 | 1.26 | 1.11 | 0.94 | 0.78 | 1.19 | 1.02 |
| *g__Granulicatella* | 2.09 | 0.86 | 1.55 | 0.92 | 2.03 | 1.56 | 1.84 | 0.95 | 1.81 | 0.97 | 1.29 | 0.56 |
| *g__Bifidobacterium* | 0.43 | 0.67 | 0.39 | 0.68 | 0.59 | 0.97 | 0.48 | 0.88 | 0.50 | 0.78 | 0.34 | 0.60 |
| *g__Fusobacterium* | 1.56 | 1.41 | 1.92 | 1.26 | 1.62 | 1.31 | 1.24 | 1.10 | 1.07 | 0.90 | 1.55 | 1.17 |
| *g__Porphyromonas* | 4.72 | 2.97 | 4.37 | 4.99 | 3.69 | 3.20 | 2.28 | 1.55 | 3.51 | 3.69 | 2.02 | 1.43 |
| *g__Capnocytophaga* | 0.66 | 0.70 | 0.73 | 0.64 | 0.78 | 0.84 | 0.32 | 0.20 | 0.39 | 0.29 | 0.55 | 0.48 |
| *g__Leptotrichia* | 0.97 | 0.83 | 1.39 | 1.93 | 1.00 | 0.75 | 0.70 | 0.65 | 0.87 | 0.79 | 0.83 | 0.85 |
| *g__Parvimonas* | 0.19 | 0.20 | 0.12 | 0.12 | 0.17 | 0.39 | 0.09 | 0.09 | 1.04 | 4.08 | 0.12 | 0.18 |
| *g__Aggregatibacter* | 0.63 | 0.90 | 0.46 | 0.53 | 0.67 | 0.60 | 0.34 | 0.31 | 0.57 | 0.52 | 0.78 | 0.80 |
| *g__Gemella* | 1.55 | 0.95 | 1.21 | 0.59 | 1.31 | 1.21 | 1.39 | 0.92 | 1.20 | 0.81 | 1.14 | 0.56 |

**Supplementary Table S2:**

**The table shows the relative abundance (%) of the core species in term (TB) and preterm (PTB) groups at each of the trimesters.**

| **Species** | **Average (%) and Standard deviation (±)** | | | | | | | | | | | |
| --- | --- | --- | --- | --- | --- | --- | --- | --- | --- | --- | --- | --- |
|  | **PTB-T1**  **(%)** | **(±) SD** | **TB-T1 (%)** | **(±) SD** | **PTB-T2 (%)** | **(±) SD** | **TB-T2**  **(%)** | **(±) SD** | **PTB-T3 (%)** | **(±) SD** | **TB-T3 (%)** | **(±) SD** |
| *s__Streptococcus symci* | 21.20 | 10.84 | 15.52 | 12.12 | 16.49 | 15.32 | 20.74 | 14.03 | 24.73 | 17.82 | 22.20 | 13.55 |
| *s__Streptococcus cristatus* | 0.84 | 0.56 | 0.81 | 0.61 | 0.93 | 0.84 | 1.80 | 1.78 | 1.73 | 1.28 | 1.55 | 1.08 |
| *s__Streptococcus toyakuensis* | 0.27 | 0.28 | 0.70 | 1.23 | 0.48 | 0.77 | 0.49 | 0.51 | 2.28 | 3.14 | 1.37 | 3.18 |
| *s__Streptococcus ilei* | 0.57 | 0.98 | 0.70 | 1.03 | 0.76 | 1.42 | 2.01 | 3.68 | 0.88 | 0.87 | 1.15 | 1.01 |
| *s__Streptococcus salivarius* | 1.06 | 2.76 | 0.56 | 0.71 | 0.57 | 0.53 | 1.99 | 2.22 | 2.12 | 3.52 | 1.71 | 2.53 |
| *s__Streptococcus rubneri* | 0.43 | 0.42 | 0.52 | 0.40 | 0.52 | 0.82 | 1.24 | 1.13 | 0.82 | 0.60 | 0.68 | 0.46 |
| *s__Streptococcus lactarius* | 0.27 | 0.21 | 0.48 | 0.41 | 0.37 | 0.48 | 0.54 | 0.60 | 0.63 | 0.49 | 0.53 | 0.40 |
| *s__Streptococcus parasanguinis* | 0.89 | 2.79 | 0.35 | 0.42 | 0.33 | 0.21 | 1.23 | 1.96 | 0.81 | 0.86 | 0.68 | 0.59 |
| *s__Streptococcus peroris* | 0.30 | 0.29 | 0.20 | 0.19 | 0.21 | 0.24 | 0.73 | 0.84 | 0.67 | 0.64 | 0.59 | 0.58 |
| *s__Streptococcus pneumoniae* | 0.12 | 0.25 | 0.09 | 0.13 | 0.12 | 0.13 | 0.37 | 1.12 | 0.15 | 0.30 | 0.59 | 1.89 |
| *s__Haemophilus parainfluenzae* | 7.83 | 6.82 | 12.84 | 7.86 | 7.08 | 6.07 | 9.62 | 9.63 | 5.67 | 6.25 | 10.43 | 8.15 |
| *s__Haemophilus sputorum* | 0.40 | 1.10 | 0.26 | 0.33 | 0.24 | 0.41 | 0.31 | 0.54 | 0.20 | 0.33 | 0.51 | 0.64 |
| *s__Neisseria perflava* | 7.12 | 7.89 | 11.33 | 12.52 | 15.77 | 15.24 | 10.46 | 10.90 | 8.63 | 8.85 | 9.13 | 9.46 |
| *s__Neisseria subflava* | 2.80 | 3.35 | 5.47 | 7.42 | 4.96 | 5.91 | 4.76 | 6.49 | 3.84 | 5.06 | 7.27 | 12.92 |
| *s__Neisseria macacae* | 0.52 | 0.56 | 0.98 | 1.52 | 1.35 | 2.83 | 0.74 | 1.35 | 1.24 | 2.03 | 0.87 | 0.96 |
| *s__Neisseria elongata* | 0.22 | 0.39 | 0.21 | 0.23 | 0.27 | 0.34 | 0.19 | 0.21 | 1.93 | 6.86 | 0.18 | 0.21 |
| *s__Veillonella nakazawae* | 3.08 | 3.19 | 4.51 | 4.45 | 5.36 | 7.93 | 3.33 | 3.72 | 2.67 | 3.71 | 3.51 | 3.93 |
| *s__Veillonella tobetsuensis* | 2.89 | 2.76 | 3.45 | 3.05 | 2.02 | 2.52 | 1.96 | 2.26 | 1.38 | 1.51 | 1.55 | 1.52 |
| *s__Veillonella atypica* | 2.24 | 4.00 | 2.31 | 3.32 | 2.77 | 7.17 | 1.80 | 2.43 | 1.64 | 3.26 | 2.26 | 3.41 |
| *s__Veillonella rogosae* | 1.14 | 1.35 | 1.43 | 1.66 | 0.64 | 1.05 | 0.91 | 1.71 | 0.41 | 0.50 | 0.56 | 0.66 |
| *s__Rothia mucilaginosa* | 10.29 | 9.88 | 3.40 | 5.61 | 6.46 | 6.58 | 7.31 | 5.60 | 7.04 | 6.81 | 5.16 | 5.15 |
| *s__Prevotella melaninogenica* | 7.55 | 5.56 | 5.25 | 4.21 | 4.05 | 5.29 | 4.44 | 4.11 | 3.19 | 4.36 | 3.67 | 4.47 |
| *s__Prevotella pallens* | 0.66 | 0.75 | 0.98 | 1.46 | 0.40 | 0.53 | 0.55 | 0.57 | 0.49 | 1.02 | 0.53 | 0.55 |
| *s__Prevotella nanceiensis* | 0.69 | 0.65 | 0.69 | 1.28 | 0.37 | 0.43 | 0.44 | 0.43 | 0.39 | 0.75 | 0.28 | 0.28 |
| *s__Prevotella histicola* | 0.73 | 1.51 | 0.43 | 0.73 | 0.43 | 1.04 | 0.63 | 0.95 | 0.46 | 1.09 | 0.38 | 0.71 |
| *s__Porphyromonas pasteri* | 3.77 | 2.88 | 3.78 | 4.73 | 2.86 | 2.52 | 1.73 | 1.21 | 2.39 | 3.28 | 1.47 | 1.37 |
| *s__Porphyromonas endodontalis* | 0.24 | 0.30 | 0.19 | 0.28 | 0.24 | 0.39 | 0.17 | 0.22 | 0.64 | 1.64 | 0.22 | 0.24 |
| *s__Leptotrichia wadei* | 0.31 | 0.44 | 0.62 | 1.46 | 0.39 | 0.48 | 0.26 | 0.40 | 0.34 | 0.55 | 0.29 | 0.60 |
| *s__Aggregatibacter aphrophilus* | 0.17 | 0.30 | 0.21 | 0.28 | 0.21 | 0.24 | 0.16 | 0.17 | 0.16 | 0.19 | 0.54 | 0.62 |
| *s__Fusobacterium pseudoperiodonticum* | 1.08 | 1.05 | 1.41 | 1.12 | 0.94 | 0.91 | 0.83 | 0.86 | 0.59 | 0.80 | 0.78 | 0.69 |
| *s__Gemella haemolysans* | 0.76 | 0.56 | 0.55 | 0.37 | 0.69 | 0.86 | 0.76 | 0.63 | 0.79 | 0.64 | 0.70 | 0.39 |
| *s__Granulicatella adiacens* | 1.68 | 0.85 | 1.29 | 0.88 | 1.67 | 1.47 | 1.49 | 0.86 | 1.37 | 0.85 | 1.07 | 0.53 |

**Supplementary Figure Legends:**

**Fig. S1:** The PCoA plot showing the distribution of (a) Bray-Curtis, (b) Jaccard indices in TB and PTB samples.

**Fig. S2:** The significant correlation plots between core microbial species and BMI. The Y-axis denotes the relative abundance of each taxon mentioned, and the X-axis denotes the BMI score. (a) The correlation plot between BMI and *Porphyromonas endodontalis*, (b) The correlation plot between BMI and *Haemophilus sputorum* in PTB group. (c) The correlation plot between BMI and *Aggregatibacter aphrophilus*, (d) The correlation plot between BMI and *Haemophilus sputorum* in TB samples.

**Fig. S3:** The results of the Random Forest Analysis showing *Rothia mucilaginosa* and *Haemophilus parainfluenzae* can predict preterm birth with 75% accuracy in the first trimester of pregnancy

**Appendix-III: Codes used in this study**

**# Code for diversity estimation in R tool**

library("vegan")

shannon_index = diversity (data, index = "shannon")

simpson_simpson = diversity (data, index = "simpson")

**# Core for box-plot in R tool**

boxplot (X1, X2, col =c("red","green"),

names = c("X1","X2"), varwidth = TRUE, ylab = "name of Y axis")

**# Code for bar plot in R tool**

A1 = data.frame(A)

a1 = as.matrix(A1)

barplot(a1,names = colnames(A1), col= 1:5,las=2,

legend = rownames(A1),ylim = c(0, 100),

xlab = "x-axis name",

ylab = "y-axis name",

main = " Barplot showing individuals data",

xlim = c(0, ncol(a1) + 25),

args.legend=list(

x=ncol(a1) + 29,

y=max(colSums(a1)+2),

bty = "n", cex = 0.7

**# Code for Hierarchical clustering with microbiome abundance data**

The species abundance table is used as input for the Hierarchical clustering dendrogram representation. R package “gplots”, “Heatplus”, “vegan”, “RColorBrewer” were used for the analysis. The distance of the data set was measured using hclust () function, the heatmap was generated by heatmap () function in R. The details of the R code used in this study was taken from “<https://www.molecularecologist.com/2013/08/20/making-heatmaps-with-r-for-microbiome-analysis/>”.

**# Code for LME (qiime2 longitudinal plugin)**

$ qiime longitudinal linear-mixed-effects --m-metadata-file metadata.tsv --m-metadata-file Rel_ab.qza' --p-metric birth_type --p-group-columns Taxon --p-state-column trimester --p-individual-id-column studyid --o-visualization linear-mixed-effect.qzv

$ qiime longitudinal plot-feature-volatility Inputs file_name --m-metadata-file metadata_file --o-visualization output_name

**# Correlation plot in R tool**

**library**("ggpubr")

ggscatter(my_data, x = "data _1", y = "data_2", add = "reg.line", conf.int = TRUE, cor.coef = TRUE, cor.method = "Spearman", xlab = "X-axis name", ylab = "Y-axis name)")

**#Code for Wilcoxon sum rank test**

A.pval= apply(A,1,function(y){

Case = as.numeric(y[1:20])

control = as.numeric(y[21:40])

res = wilcox.test (case, control, alternative = c("two.sided"), paired=FALSE)

return(res$p.value)

})

**# Code for Random Forest (RF) analysis**

library(randomForest)

library(caret)

library(caTools)

library(verification)

library(pROC)

library(ggthemes)

library(grid)

library(gridExtra)

phsc <- read.table("species-data.txt", h = T, sep = "\t")

phsc$Group <- as.factor(phsc$Group)

\# spliting and define training and testing dataset

df <- phsc[,2:36]

spl <- sample.split(df$Group, SplitRatio = 0.6)

Train <- subset(df, spl == TRUE)

Test <- subset(df, spl == FALSE)

Train$Group <- as.factor(Train$Group)

Test$Group <- as.factor(Test$Group)

##prediction model build-up using train dataset

random_forest=randomForest(Group~.,data=Train,ntree=1000,importance=TRUE)

random_forest

##identified importance variables from the prediction model

imp=importance(random_forest)

impL=imp[,c(4,5)]

imp.ma=as.matrix(impL)

imp.df=data.frame(imp.ma)

\## write down the importance variables in a text file

write.csv(imp.df, "imp.case-control.csv", row.names=TRUE)

\### plot the variable importance data ###

##read that file

imp.df.csv=read.csv("imp.case-control.csv",header=TRUE)

colnames(imp.df.csv)=c("Variable","MeanDecreaseAccuracy","MeanDecreaseGini")

imp.sort = imp.df.csv[order(-imp.df.csv$MeanDecreaseAccuracy),]

imp.sort = transform(imp.df.csv,

Variable = reorder(Variable, MeanDecreaseAccuracy))

VIP=ggplot(data=imp.sort, aes(x=Variable, y=MeanDecreaseAccuracy)) +

ylab("Mean Decrease Accuracy")+xlab("")+

geom_bar(stat="identity",fill="skyblue",alpha=.8,width=.75)+

coord_flip()+theme_few()

imp.sort.Gini <- transform(imp.df.csv,

Variable = reorder(Variable, MeanDecreaseGini))

VIP.Gini=ggplot(data=imp.sort.Gini, aes(x=Variable, y=MeanDecreaseGini)) +

ylab("Mean Decrease Gini")+xlab("")+

geom_bar(stat="identity",fill="skyblue",alpha=.8,width=.75)+

coord_flip()+theme_few()

VarImpPlot=arrangeGrob(VIP)

\# draw plot

tiff("mean_decrease_plot_three_group_1.tiff", width = 5, height = 10, units = 'in', res = 300)

grid.draw(VarImpPlot)

dev.off ()
